# Implanting toric implantable collamer lens displays better astigmatism correction than implantable collamer lens combined with manually limbal relaxing incision

**DOI:** 10.1186/s12886-023-02941-1

**Published:** 2023-05-05

**Authors:** Ke Yang, Jiaxin Li, Weihua Zhang, Zhanjiang Liu, Chenjie Song, Yang Zhao

**Affiliations:** 1 Beijing Tongren Eye Center, Beijing Key Laboratory of Ophthalmology and Visual Science, Beijing Tongren Hospital, Capital Medical University, Beijing, 100730 China; 2 Medical School, Pingdingshan University, Pingdingshan, 467000 China; 3 Liaocheng People’s Hospital, Liaocheng, 252000 China; 4 Chaoyang Central Hospital, Liaoning Province, 122000 China

**Keywords:** Implantable collamer lens, Limbal relaxing incision, Myopia, Astigmatism, Vision care

## Abstract

**Background:**

This retrospective study aimed to compare the outcomes of toric implantable collamer lens (TICL) surgery with those of implantable collamer lens (ICL) implantation combined with limbal relaxing incision (LRI) in patients with low myopia and astigmatism.

**Methods:**

A total of 40 eyes of 28 patients who underwent TICL implantation and 40 eyes of 27 patients who underwent ICL implantation combined with manually LRI between 2021 and 2022 were included. Primary outcomes were manifest sphere and cylinder, intraocular pressure, visual acuity, and astigmatism parameters at 1 day, 1 week, and 1, 3, and 6 months postoperatively.

**Results:**

The two surgeries showed comparable effects on manifest sphere and cylinder, intraocular pressure, and visual acuity (all p > 0.1). Surgery-induced astigmatism (SIA) was maintained as stable in the TICL group (1.73 to 1.68, p = 0.420), but was significantly reduced in the ICL/LRI group (1.74 to 1.17, p = 0.001) from preoperative to postoperative 6 months. The TICL group displayed significantly higher SIA and correction index at postoperative 1, 3, and 6 months than the ICL/LRI group (at 6 months: SIA, 1.68 (1.26, 1.96) vs., 1.17 (1.00, 1.64), p = 0.010; CI: 0.98 (0.78, 1.25) vs. 0.80 (0.61, 1.04), p = 0.018). No complications occurred during follow-up.

**Conclusions:**

The effects of ICL/LRI are comparable to those of TICL in correcting myopia. TICL implantation displays better astigmatism correction than ICL/LRI.

**Supplementary Information:**

The online version contains supplementary material available at 10.1186/s12886-023-02941-1.

## Background

Various techniques have been developed to treat myopia and prevent wearing eyeglasses or contact lenses. An implantable collamer lens (ICL) was first successfully implanted into the eye of a 45-year-old female in 1949 [1]. Since then, ICLs have been used for cataract treatment and adjusting refractive errors such as myopia and hyperopia. However, astigmatism still requires correction in some patients with ICL implantation. In 1999, the first toric implantable collamer lens (TICL) was implanted [2, 3]. TICL improves ICL by adding the ability to manifest cylinder, thus allowing the ICL to adjust myopia and astigmatism simultaneously.

The implantation procedures for ICL and TICL are identical except for one extra step in implanting TICL [4–6]. In both myopia and astigmatism, the cornea is incised, the folded lens is inserted through the incision, and the lens is unfolded and placed in position. To achieve effective correction of astigmatism, the surgeon must achieve exact alignment of the cylinder axis of TICL and the axis of corneal astigmatism. Thus, after positioning the TICL, the lens needs to be rotated for alignment. Theoretically, the rotated TICL will stay that way forever. But TICL has a 2% pooled incidence rate of repositioning surgery [7]. This means that some patients with TICL implantation need to go back to the operating room to restore their visual acuity. Thus, a newly designed TICL that can eliminate the need for repositioning surgery, or new procedures of refractive surgery that can fix astigmatism in ICL implanted patients is needed.

Like arcuate keratectomy (AK), limbal relaxing incision (LRI) is one of the methods used to correct astigmatism [8, 9]. In both surgeries, the cornea is incised at both sides of the steep axis of corneal astigmatism. This relaxes the steep axis of corneal astigmatism while steepening the flat axis. In turn, this changes the shape of the cornea and corrects astigmatism. Unlike AK, the incisions are performed more centrally in the cornea, the incision sites of LRI are adjacent to the limbus and just anterior to the vascular arcade. Compared with AK, LRI is more predictable, with less discomfort and fewer complications in correcting astigmatism [9].

Since TICL and ICL combined with LRI (ICL/LRI) can correct both myopia and astigmatism, it is interesting to compare results of the two operations. This study aimed to compare the outcomes of TICL and ICL/LRI in patients with low myopia and astigmatism.

## Materials and methods

This study enrolled subjects diagnosed with myopia and astigmatism who underwent TICL surgery or ICL combined with LRI surgeries at Beijing Tongren Hospital between September 2021 and September 2022. Inclusion criteria were: (1) patients who decided to accept ICL surgery; (2) had astigmatism less than 2.0D; (3) had differences between the steepest meridian of astigmatism and the corneal K2 axis of less than 10°; (4) aged between 18 and 50 years; or (5) with regular astigmatism (1D-2.5D). Patients with cataract, glaucoma, or fundus diseases were excluded. Patients were randomly assigned to the ICL/LRI group or the TICL group. All surgeries were performed independently by the same team.

### Ethics statement

The study followed the Declaration of Helsinki. The study protocol was reviewed and approved by the internal review board (IRB) of Beijing Tongren Hospital, and all patients provided signed informed consent at the time of surgery for their perioperative data to be recorded for subsequent study and publication.

### TICL surgical procedure

Patients remained in a sitting position under the slit lamp to mark the horizontal point of the cornea. The patients then moved onto the operating table and lay flat. The cornea was marked with a 1 mL needle tip to label the placement axis for TICL. The main 3-millimeter incision was made at 180° and a 1-millimeter side incision was made with an 15° incision knife (MANI Inc. Japan) at another angle to allow position adjusting for TICL. After implantation, the TICL cylinder axis was aligned with the axis of astigmatism.

The TICL and water-filled IA were inserted through the main incision, and the implantable lens adjustment hook was rotated and moved the TICL through the side incision. After the anterior chamber was maintained and the four haptics of the TICL were moved under the iris, the wound was cleaned using sterile saline. Patients were then sent to the recovery room.

### LRI-related calculation

Patients’ optometric data were entered into the LRI surgical planning software (www.lricalculator.com) to get the incision site and length. The surgically induced cylinder was set at 0.4D. The calculated results were recorded. The incision length was also calculated by the equation developed by the author. In the equation, astigmatism in D was multiplied by 100 and divided by 2. For example, if a patient needs to release 1.6D astigmatism, the astigmatism release arc length is 1.6*100/2 = 80°. The maximum is 90°, with 10° flexibility for the operation. The equation was shown to provide similar results as the online software.

### ICL/LRI surgical procedure

Patients remained in the seated position under the slit lamp to mark the horizontal axis. The patients then moved onto the operating table and lay flat. The starting and ending points of the proposed incision on the cornea were marked at both sides of the steep meridian of astigmatism with the help of a Mendez-style ring. A patented limbal release auxiliary device was used to assist the LRI surgery. The device was placed on the eyeball and the cornea was put in the center of the device. At the moment of incision, the device was slightly pressed to fix the eyeball. Based on the measured thickness of the cornea, an LRI knife with a suitable depth (MANI Inc. Japan) was used to cut into about 90% corneal depth. With the help of the auxiliary device, a vertical to the surface and curved incision can be made. The main incision with the desired length was made at the opposite side of the first LRI. The ICL and water-filled IA were inserted through the main incision, and the implantable lens adjustment hook also helped to move the ICL through the main incision. After the anterior chamber was maintained and the four haptics of the ICL were moved under the iris, the wound was cleaned using sterile saline. Patients were then sent to the recovery room.

### Routine examination

Patients were examined preoperatively and 1 day, 1 week, 1 month, 3 months, and 6 months after surgery. The same optometrist conducted the ophthalmic examination for the patient throughout the whole follow-up procedure. Manifest sphere, Manifest cylinder, intraocular pressure, visual acuity (logMAR), and astigmatism. The CI is the ratio of surgical-induced astigmatism (SIA) to target-induced astigmatism (TIA). A correction index (CI) value > 1 (< 1) means RA overcorrection (undercorrection).

### Statistical analysis

We used G*Power 3.1.9.7 software for power analysis with the following settings: MANOVA: Repeated measures, within-between interaction,α = 0.05, power = 0.8, group = 2, number of measurement = 5, and effect size = 0.4. The calculated sample size was 80.

Continuous data with normal distribution are presented as mean ± standard deviation (SD) and analyzed by Student’s t test; Continuous data without normal distribution are presented as median (interquartile range, IQR) and analyzed by the Wilcoxon rank sum test; Categorical data are presented as n (%) and analyzed by the chi-squared test or Fisher exact test, as appropriate. Generalized estimating equations (GEE) with unstructured matrix were performed to evaluate the association between the surgery type and the postoperative performance of manifest sphere, manifest cylinder, intraocular pressure, and visual acuity. Stratified analyses were performed to observe the difference between the left and right eyes. Vector analysis within different time were using Friedman’s test and presented as median (IQR) because of data without normal distribution. All p values were two-sided and p < 0.05 was considered statistically significant. All statistical analyses were performed using the statistical software package SAS software version 9.4 (SAS Institute Inc., Cary, NC, USA).

## Results

A total of 80 eyes of 55 patients were included. The average age was 28.36 ± 6.09, and women accounted for 70% of patients. The TICL group contained 40 eyes of 28 patients and the ICL/LRI group contained 40 eyes of 27 patients.

### Visual outcome analysis

Table 1 shows the preoperative characteristic of 80 eyes in ICL/LRI and TICL groups. No significant differences were found in age, sex, myopia, astigmatism, and visual acuity at baseline between ICL/LRI and TICL groups. The same results can be observed when the analysis is stratified by eye, as shown in Table 2.

**Table 1:** Preoperative characteristics of 80 eyes between ICL/LRI and TICL groups

Variable	Total (n = 80)	Surgery		
		ICL/LRI (n = 40)	TICL (n = 40)	*p*-value
Age	28.36 ± 6.09	28.98 ± 6.89	27.75 ± 5.18	0.372
Sex				1.000
Female	56 (70.00)	28 (70.00)	28 (70.00)	
Male	24 (30.00)	12 (30.00)	12 (30.00)	
Before surgery				
Manifest sphere	-8.50 (-10.00, -6.88)	-8.75 (-10.00,-6.75)	-8.13 (-10.00,-7.00)	0.214
Manifest cylinder	-1.50 (-1.75, -1.25)	-1.25 (-1.50, -1.25)	-1.75 (-1.75, -1.50)	0.124
Corrected visual acuity	0.00 (0.00, 0.00)	0.00 (0.00, 0.00)	0.00 (0.00, 0.00)	0.881

Continuous data with normal distribution are presented as mean ± SD and analyzed by Student’s t test; Continuous data without normal distribution are presented as median (IQR) and analyzed by the Wilcoxon rank sum test; Categorical data are presented as n (%) and analyzed by the chi-squared test

**Table 2:** Preoperative characteristics of 80 eyes between ICL/LRI and TICL groups in different eyes

Variable	Left				Right			
	Total (n = 40)	Surgery			Total (n = 40)	Surgery		
		ICL/LRI (n = 18)	TICL (n = 22)	*p*-value		ICL/LRI (n = 22)	TICL (n = 18)	*p*-value
Age	28.58 ± 6.13	29.44 ± 7.25	27.86 ± 5.11	0.424	28.15 ± 6.12	28.59 ± 6.74	27.61 ± 5.41	0.621
Sex				0.919				1.000^a^
Female	27 (67.50)	12 (66.67)	15 (68.18)		29 (72.50)	16 (72.73)	13 (72.22)	
Male	13 (32.50)	6 (33.33)	7 (31.82)		11 (27.50)	6 (27.27)	5 (27.78)	
Before surgery								
Manifest sphere	-8.69 ± 2.83	-9.15 ± 3.70	-8.32 ± 1.85	0.392	-8.74 ± 3.14	-9.34 ± 3.75	-8.01 ± 2.04	0.164
Manifest cylinder	-1.50 (-1.75, -1.25)	-1.25 (-1.50, -1.25)	-1.75 (-1.75, -1.50)	0.076	-1.50 (-1.75, -1.25)	-1.25 (-1.75, -1.25)	-1.75 (-2.00, -1.50)	0.578
Corrected visual acuity	0.00 (0.00, 0.00)	0.00 (0.00, 0.00)	0.00 (0.00, 0.11)	0.740	0.00 (0.00, 0.00)	0.00 (0.00, 0.00)	0.00 (0.00, 0.00)	0.758

Continuous data with normal distribution are presented as mean ± SD and analyzed by Student’s t test; Continuous data without normal distribution are presented as median (IQR) and analyzed by the Wilcoxon rank sum test; Categorical data are presented as n (%) and analyzed by the chi-squared test or Fisher exact test^a^

**Fig. 1:**
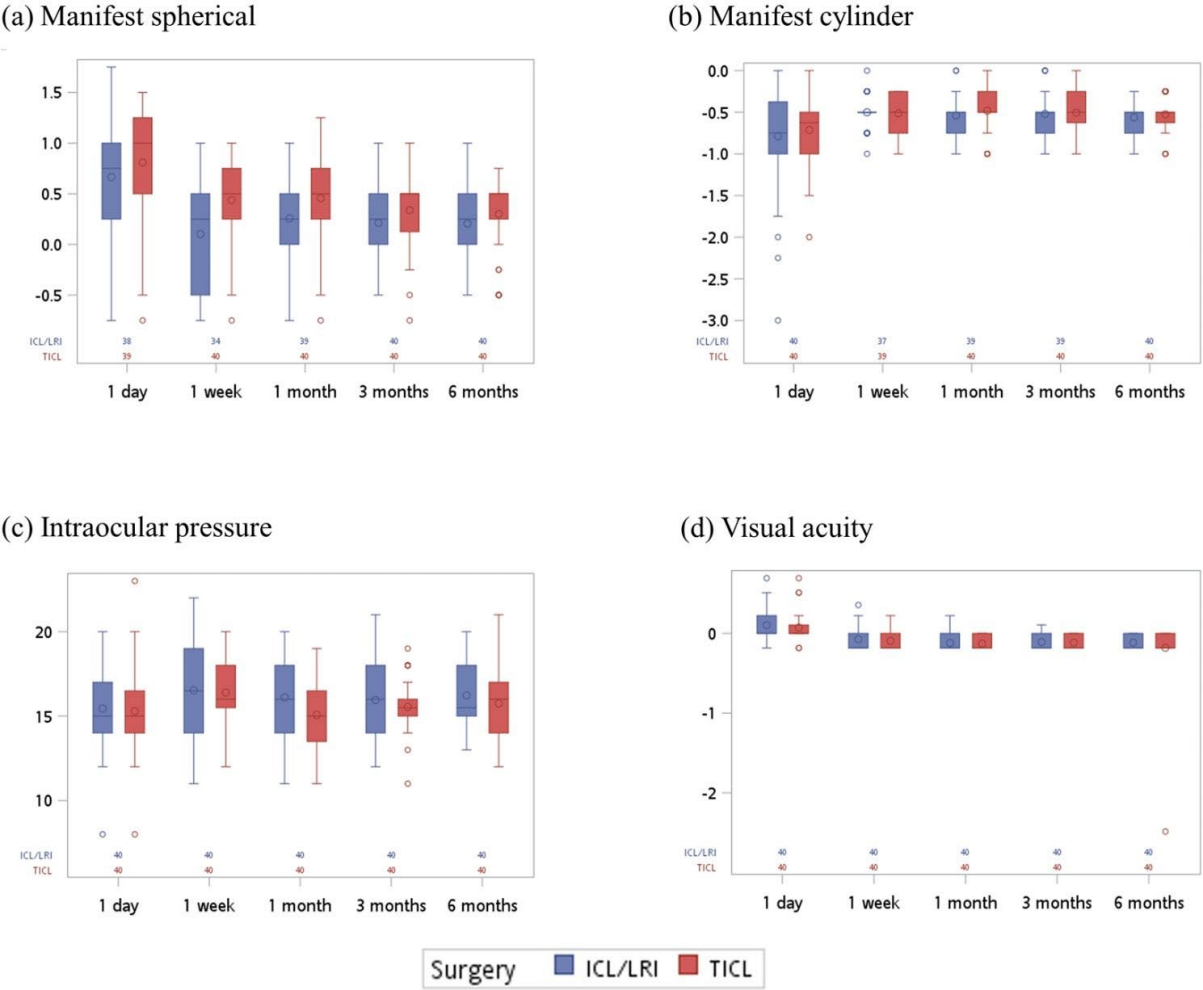
Line charts of the results of TICL or ICL/LRI surgery at different observed time points in (**a**) manifest sphere, (**b**) manifest cylinder, (**c**) intraocular pressure, and (**d**) visual acuity. The data in the chart are presented as mean ± SD.

Figure 1 and Supplementary Table S1 show that the value of manifest sphere in the ICL/LRI group was closer to normal (value = 0) (median of manifest sphere: ICL/LRI: 0.25 vs. TICL:0.50, p = 0.006 and 0.039) at the first postoperative week and 1 month after surgery. Supplementary Figures S1 and S2 and Supplementary Tables S2 and S3 show the results after stratification. In the right eye, the value of manifest sphere at the first week after surgery was relatively normal in the ICL/LRI group (median of manifest sphere: ICL/LRI: -0.25 vs. TICL:0.50, p = 0.019).

Table 3 shows the associations between the surgery and the postoperative performance of manifest sphere, manifest cylinder, intraocular pressure, and visual acuity. The values of manifest sphere were significantly lower at 1 week to 6 months postoperatively compared to the first postoperative day (β: -0.65 to -0.44). No significant differences were noted between the two procedures (p = 0.317). In the manifest cylinder, the values were significantly higher at 1 week, 3 months, and 6 months postoperatively compared to the first day (p = 0.03, 0.02, 0.047, respectively), and no significant differences were found between the two procedures (p = 0.525). Regarding intraocular pressure, compared with the first day, the values were significantly higher at 1 week and 1 month postoperatively (p = 0.005 and 0.049), and no significant differences were found between three and six months postoperatively and the day after the operation (p = 0.197 and 0.053).No significant differences were noted between the two surgical methods (p = 0.901). For visual acuity, the LogMAR values from 1 week to 6 months after surgery were significantly lower (β: -0.18 to -0.22, all p < 0.001) compared with the first day. No significant differences were found between the two surgical methods (p = 0.845). No interactions between time and groups were observed in each analysis.

**Table 3:** Associations between the surgery and postoperative performance of manifest sphere, manifest cylinder, intraocular pressure, and visual acuity

Variable	Manifest sphere			Manifest cylinder			Intraocular pressure			Visual acuity		
	β (95% CI)	*p*-value		β (95% CI)	*p*-value		β (95% CI)	*p*-value		β (95% CI)	*p*-value	
Age	0.04 (-0.03, 0.11)	0.267		0.00 (-0.01, 0.01)	0.520		0.20 (-0.12, 0.51)	0.217		0.02 (-0.01, 0.04)	0.162	
Male (vs. female)	-0.84 (-1.73, 0.06)	0.067		0.02 (-0.18, 0.22)	0.858		2.35 (-0.82, 5.52)	0.147		-0.03 (-0.19, 0.14)	0.766	
Surgery (vs. TICL)												
ICL/LRI	0.21 (-0.20, 0.62)	0.317		0.07 (-0.15, 0.29)	0.525		0.09 (-1.31, 1.49)	0.901		-0.01 (-0.09, 0.07)	0.845	
Time (vs. 1 day)												
1 week	**-0.65 (-0.98, -0.33)**	**< 0.001**		**0.39 (0.04, 0.75)**	**0.030**		**1.08 (0.32, 1.83)**	**0.005**		**-0.18 (-0.23, -0.13)**	**< 0.001**	
1 month	**-0.52 (-0.88, -0.16)**	**0.004**		0.19 (-0.07, 0.45)	0.150		**0.65 (0.002, 1.30)**	**0.049**		**-0.22 (-0.28, -0.16)**	**< 0.001**	
3 months	**-0.44 (-0.62, -0.25)**	**< 0.001**		**0.26 (0.04, 0.48)**	**0.020**		0.50 (-0.26, 1.26)	0.197		**-0.21 (-0.28, -0.15)**	**< 0.001**	
6 months	**-0.44 (-0.62, -0.26)**	**< 0.001**		**0.22 (0.00, 0.45)**	**0.047**		0.78 (-0.01, 1.56)	0.053		**-0.22 (-0.29, -0.14)**	**< 0.001**	
Time x Surgery (vs. 1 day, TICL)												
1 week	0.28 (-0.09, 0.65)	0.139		0.10 (-0.21, 0.42)	0.526		0.02 (-1.04, 1.09)	0.963		0.01 (-0.07, 0.09)	0.798	
1 month	0.17 (-0.24, 0.57)	0.416		0.02 (-0.32, 0.37)	0.890		-0.88 (-1.79, 0.04)	0.060		0.02 (-0.06, 0.11)	0.623	
3 months	-0.04 (-0.29, 0.21)	0.775		0.10 (-0.25, 0.44)	0.587		-0.25 (-1.45, 0.95)	0.683		0.03 (-0.06, 0.11)	0.540	
6 months	-0.07 (-0.33, 0.19)	0.610		0.05 (-0.28, 0.38)	0.767		-0.33 (-1.50, 0.85)	0.588		-0.04 (-0.18, 0.11)	0.622	

Presented as β (95%CI) and analyzed by generalized estimating equations (GEE) with unstructured matrix. Significant values are shown in bold

Supplementary Tables S4 and S5 show the associations between the surgery type and postoperative performance in the left and right eyes. Analysis of the left eye (Table S4) showed that the manifest cylindrical value was significantly higher only at 1 week but not at the times over 1 month postoperatively compared to the first postoperative day (p = 0.012), and no significant differences were found between the two procedures (p = 0.798). The intraocular pressure was significantly higher at 6 months postoperatively than on the first postoperative day (p = 0.031), and no significant differences were found between the two procedures (p = 0.22). Other results are the same as the original analysis of both eyes.

In the right eye (Table S5), no significant differences were observed in all outcomes between the two types of surgery. No interactions were observed between times and groups. The values of manifest sphere were significantly lower at 3 months and 6 months postoperatively than on the first postoperative day (p = 0.04 for both). The intraocular pressure was significantly higher at one week after surgery (p = 0.003), and no significant differences were noted between the two procedures (p = 0.931). For visual acuity, LogMAR values were significantly lower at 1 week to 6 months after the first postoperative day (β: -0.17 to -0.22, all p < 0.001).

### Corneal vector analysis of astigmatism

Table 4 shows the postoperative changes in the corneal vectors analyzed using the Alpins method. No significant differences were found between groups in target-induced astigmatism in either the TICL or ICL/LRI group (1.75 (1.38, 1.75) vs. 1.50 (1.25, 1.75), p = 0.125). The TICL group displayed significantly higher surgically-induced astigmatism (SIA) than the ICL/LRI group at 1 (1.69 (1.31, 1.98) vs. 1.26 (1.00, 1.56), p = 0.009), 3 (1.65 (1.26, 1.94), p = 0.021), and 6 months (1.68 (1.26, 1.96) vs. 1.17 (1.00, 1.64), p = 0.010). The TICL group also showed significantly better correction index (CI) than the ICL/LRI group at 1 (1.00 (0.85, 1.18) vs. 0.89 (0.65, 1.05), p = 0.035) and 6 months (0.98 (0.78, 1.25) vs. 0.80 (0.61, 1.04), p = 0.018). SIA and CI within different time had significant difference in ICL/LRI group (p = 0.001), but not found the significance in TICL group (p = 0.420). Furthermore, the corneal vectors analyses were conducted on left and right eyes. The significant difference between two groups were found at 1 month of SIA of left-eye (p = 0.046) and at 6 months of SIA and CI of right-eye (p = 0.049 and p = 0.025). The significance of SIA and CI within different time were found in right-eye ICL/LRI group, but not found in right-eye TICL group and left-eye two procedure groups.

**Table 4:** Vector analysis of postoperative astigmatism between TICL and ICL/LRI groups

Time	ICL/LRI	TICL	p-value^1^
TIA	1.50 (1.25,1.75)	1.75 (1.38,1.75)	0.125
SIA			
1 day	1.74 (1.40,1.98)	1.73 (1.45,2.14)	0.641
1 week	1.34 (1.11,1.92)	1.75 (1.31,1.97)	0.082
1 month	1.26 (1.00,1.56)	1.69 (1.31,1.98)	**0.009**
3 months	1.21 (1.00,1.75)	1.65 (1.26,1.94)	**0.021**
6 months	1.17 (1.00,1.64)	1.68 (1.26,1.96)	**0.010**
p-value^2^	**0.001**	0.420	
CI			
1 day	1.10 (1.00,1.39)	1.09 (0.96,1.28)	0.491
1 week	1.00 (0.71,1.26)	1.02 (0.88,1.18)	0.56
1 month	0.89 (0.65,1.05)	1.00 (0.85,1.18)	**0.035**
3 months	0.84 (0.67,1.05)	1.00 (0.80,1.20)	0.089
6 months	0.80 (0.61,1.04)	0.98 (0.78,1.25)	**0.018**
p-value^2^	**0.001**	0.420	
Left-eye			
TIA	1.50 (1.25, 1.50)	1.75 (1.25, 1.75)	0.078
SIA			
1 day	1.53 (1.25, 1.92)	1.77 (1.36, 2.17)	0.259
1 week	1.37 (1.04, 1.83)	1.74 (1.04, 1.97)	0.377
1 month	1.18 (1.00, 1.53)	1.69 (1.20, 2.00)	**0.046**
3 months	1.17 (1.01, 1.60)	1.57 (1.20, 1.98)	0.125
6 months	1.14 (1.02, 1.53)	1.66 (1.07, 1.97)	0.079
p-value^2^	0.270	0.676	
CI			
1 day	1.03 (0.94, 1.28)	1.09 (0.91, 1.33)	0.989
1 week	0.97 (0.82, 1.28)	1.02 (0.67, 1.16)	0.817
1 month	0.88 (0.67, 1.02)	1.04 (0.72, 1.22)	0.150
3 months	0.88 (0.72, 1.07)	0.97 (0.72, 1.18)	0.550
6 months	0.80 (0.68, 1.02)	0.95 (0.72, 1.27)	0.334
p-value^2^	0.270	0.676	
Right-eye			
TIA	1.50 (1.25, 1.75)	1.63 (1.50, 1.75)	0.588
SIA			
1 day	1.85 (1.46, 2.00)	1.69 (1.48, 1.96)	0.714
1 week	1.34 (1.23, 1.97)	1.77 (1.39, 1.97)	0.109
1 month	1.31 (1.00, 1.83)	1.62 (1.34, 1.94)	0.131
3 months	1.25 (1.00, 1.75)	1.74 (1.42, 1.91)	0.054
6 months	1.21 (1.00, 1.83)	1.70 (1.34, 1.92)	**0.049**
p-value^2^	**0.007**	0.724	
CI			
1 day	1.13 (1.00, 1.46)	1.09 (0.98, 1.25)	0.438
1 week	1.00 (0.70, 1.23)	1.02 (0.93, 1.30)	0.289
1 month	0.92 (0.63, 1.08)	1.01 (0.88, 1.15)	0.112
3 months	0.82 (0.63, 1.04)	1.02 (0.88, 1.21)	0.075
6 months	0.79 (0.60, 1.08)	1.00 (0.94, 1.24)	**0.025**
p-value^2^	**0.007**	0.724	

1: Wilcoxon rank sum test

2: Friedman’s test

Note: data are presented as median (IQR)

TIA, target induced astigmatism; SIA, surgically induced astigmatism; CI, correction index = SIA/TIA

## Discussion

The present study showed comparable effects of myopia corrections in TICL and ICL/LRI surgeries in patients with low myopia, while TICL displayed better astigmatism correction compared to ICL/LRI in patients with low astigmatism.

Vector analysis revealed the following information about outcomes. First, the differences between SIA and TIA revealed that neither TICL nor ICL/LRI achieves a perfect astigmatism correction. Second, the median of CI at all the observed time points in the TICL group remained stable over the course of 6 months, and had better CIs. Both TICL and ICL/LRI surgery can maintain its therapeutic effects within 6 months, although TICL displays better astigmatism correction than does ICL/LRI.

The observed time points in the present study were from the first postoperative day to 6 months after surgery. Compared with other studies, the starting point of observation was much closer to the date of the operation [6, 10–13]. This provides clinical evidence of changes in corneal astigmatism postoperatively. Results of vector analysis showed that, SIA dropped in the ICL/LRI group from 1.74D on postoperative day 1 to 1.17D at 6 months postoperatively. The biggest difference was in the first week when SIA changed from 1.74D to 1.34D, followed by 1.26D at 1 month. Our results confirmed that the shape of the cornea and the degree of astigmatism are not changed immediately after LRI surgery. Although the cornea is relaxed quickly in the first several days after LRI surgery, the shape of the cornea takes at least a month to stabilize. The significant reduction of SIA in the LRI group over time indicates a tendency to lose the astigmatic correction effect and for under-correction over time. Since the last follow-up was 6 months after surgery, we cannot confirm how long the effect of LRI will endure. Eliwa et al. [10] indicated that the effect of LRI was stable between 10 weeks to 3 years. This is likely applicable to outcomes of our study as well.

Myopia and astigmatism need to be minimal to achieve sufficient visual acuity. These emmetropias can be corrected by various procedures besides wearing eyeglasses. For astigmatism, procedures such as arcuate keratotomy (AK), clear corneal incisions (CCIs) along the steep meridian, opposite CCIs, limbal relaxing incisions (LRI), photorefractive keratectomy (PRK), laser-assisted in situ keratomileusis (LASIK), and TICL can be applied [14–16]. Although they have a high success rate, LASIK and PRK generate irreversible damage to the eye and are not suitable for all patients [17]. AK, CCIs, and opposite CCIS have limited predictability and often result in overcorrection and complications, especially in eyes with low and moderate astigmatism [6, 16]. Although TICL is a good choice, the waiting time for lens manufacturing is long, with a 2% chance of repositioning surgery, and the cost is much higher than other treatments. Thus, the LRI procedure, which provides earlier stability in postoperative vision and may carry a lower risk of inducing glare and discomfort, becomes more frequently performed for treatment of astigmatism at the time of cataract surgery [18, 19].

Generally, patients’ corneas will be cut three times in ICL/LRI operation. In the present study, patients with less than 10° difference between the steepest meridian of astigmatism and the corneal K2 axis were included. A 10° difference can be considered the same in ophthalmological surgery. With this requirement, one LRI could be used as the ICL insertion site in our study. This reduced the number of incisions to two rather than three, which shortened the time for surgery and recovery. Based on our observation data for 40 eyes undergoing ICL/LRI, this procedure is acceptable.

The auxiliary device used in LRI is worthy of mention. Although anesthetic eye drops and an eyelid holder were used for the LRI operation, the eyeball is still able to move around consciously. An unfixed eyeball increases the complexity and difficulty of the surgery. However, with this auxiliary device, the surgeons can press the eyeball slightly to fix its location when making an incision. This prevents the unnecessary failure of LRI procedures. Besides, the curvature of LRI is important for reaching optimal results. With this patented device, the surgeons can easily make a smooth curved incision rather than a jagged or straight line. A smooth curved incision then guarantees that the tension on the surface of the cornea is relaxed evenly. This will lead to optimal results of LRI operations with few side effects.

The present study has several limitations. First, the retrospective study design and single-center nature of the study does not allow generalization to other populations. Second, our study focused on patients with regular astigmatism and less than 10° difference between the steepest meridian of astigmatism and the corneal K2 axis. Patients who were excluded may have different results if applying the same procedure. Third, LRI is only good at adjusting minor astigmatism. Consequently, only patients with astigmatism less than 2.0D were included in the present study. Patients with a higher degree of astigmatism may not be able to be treated with ICL/LRI. TICL might be the only option for such patients. Fourth, the use of this old technique that has been largely substituted by femtosecond laser-assisted incisions. Lastly, the follow-up time was only 6 months, which may not have allowed observation of complications and the long-term effects of TICL and ICL/LRI.

## Conclusion

In patients with low myopia and low astigmatism, the ICL/LRI surgery has comparable effects to TICL in correcting myopia. TICL displays better astigmatism correction than ICL/LRI.

## Electronic supplementary material

Below is the link to the electronic supplementary material.

Supplementary Material 1

## Data Availability

All data generated during this study are included in this published article and its supplementary information files.

## List of Abbreviations

TICL: Toric implantable collamer lens
ICL/LRI: Incision and implantable collamer lens
AK: Arcuate keratectomy
LRI: Limbal relaxing incision
