# Supplementary material for: Implanting toric implantable collamer lens displays better astigmatism correction than implantable collamer lens combined with manually limbal relaxing incision
Source: BMC Ophthalmol. 2023 May 5;23:198. doi: 10.1186/s12886-023-02941-1 (PMC10161410; doi:10.1186/s12886-023-02941-1)
Supplement: Supplementary file 1 — Supplementary Material 1 [file 12886_2023_2941_MOESM1_ESM.docx]

**Supplement Figure**

**Figure S1.** Line charts of the results of TICL or ICL/LRI surgery on left eyes at different observed time points in (a) manifest sphere, (b) manifest cylinder, (c) intraocular pressure, and (d) visual acuity. The data in the chart are presented as mean ± SD.


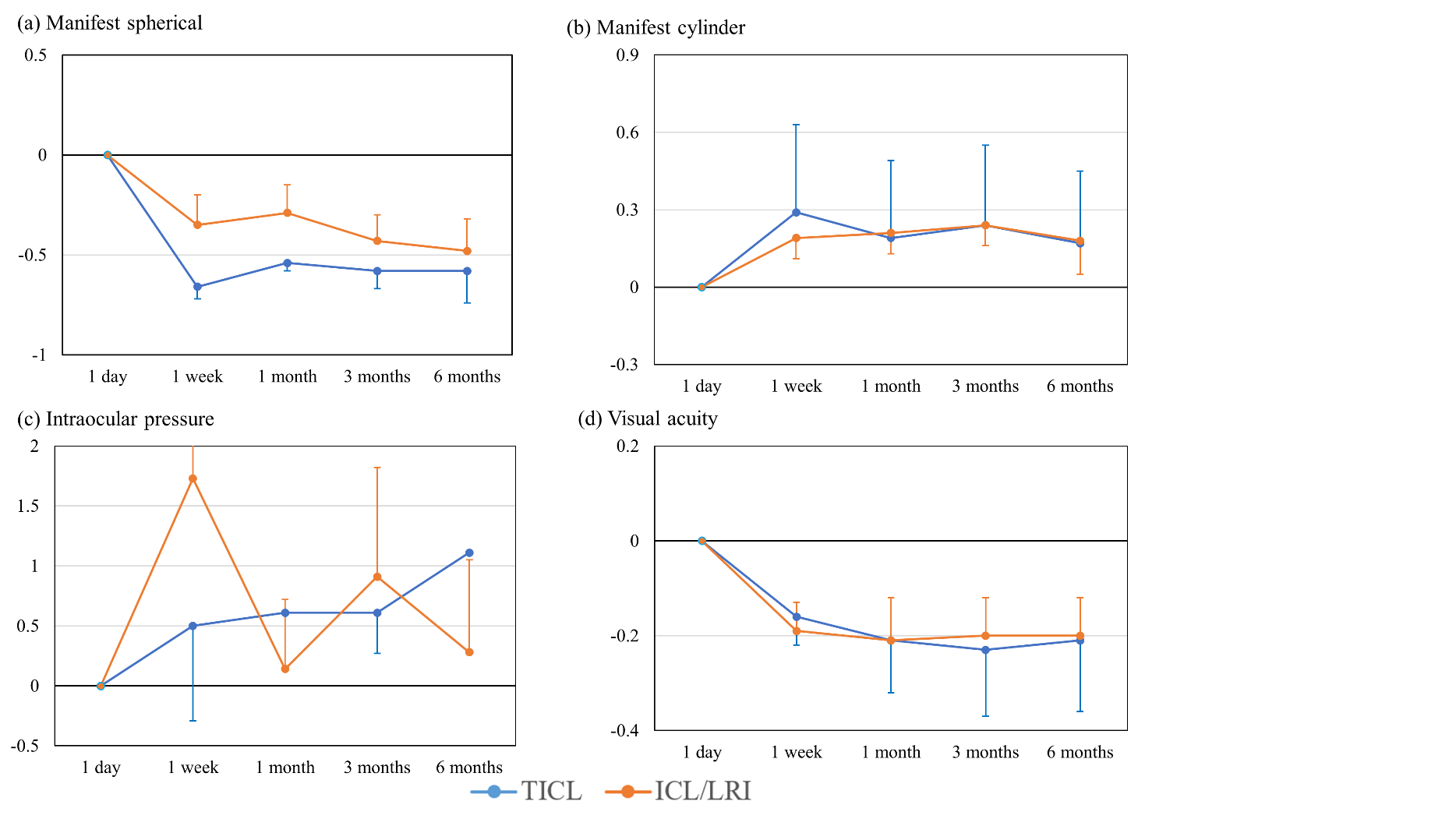


**Figure S2.** Line charts of the results of TICL or ICL/LRI surgery on right eyes at different observed time points in (a) manifest sphere, (b) manifest cylinder, (c) intraocular pressure, and (d) visual acuity. The data in the chart are presented as mean ± SD.


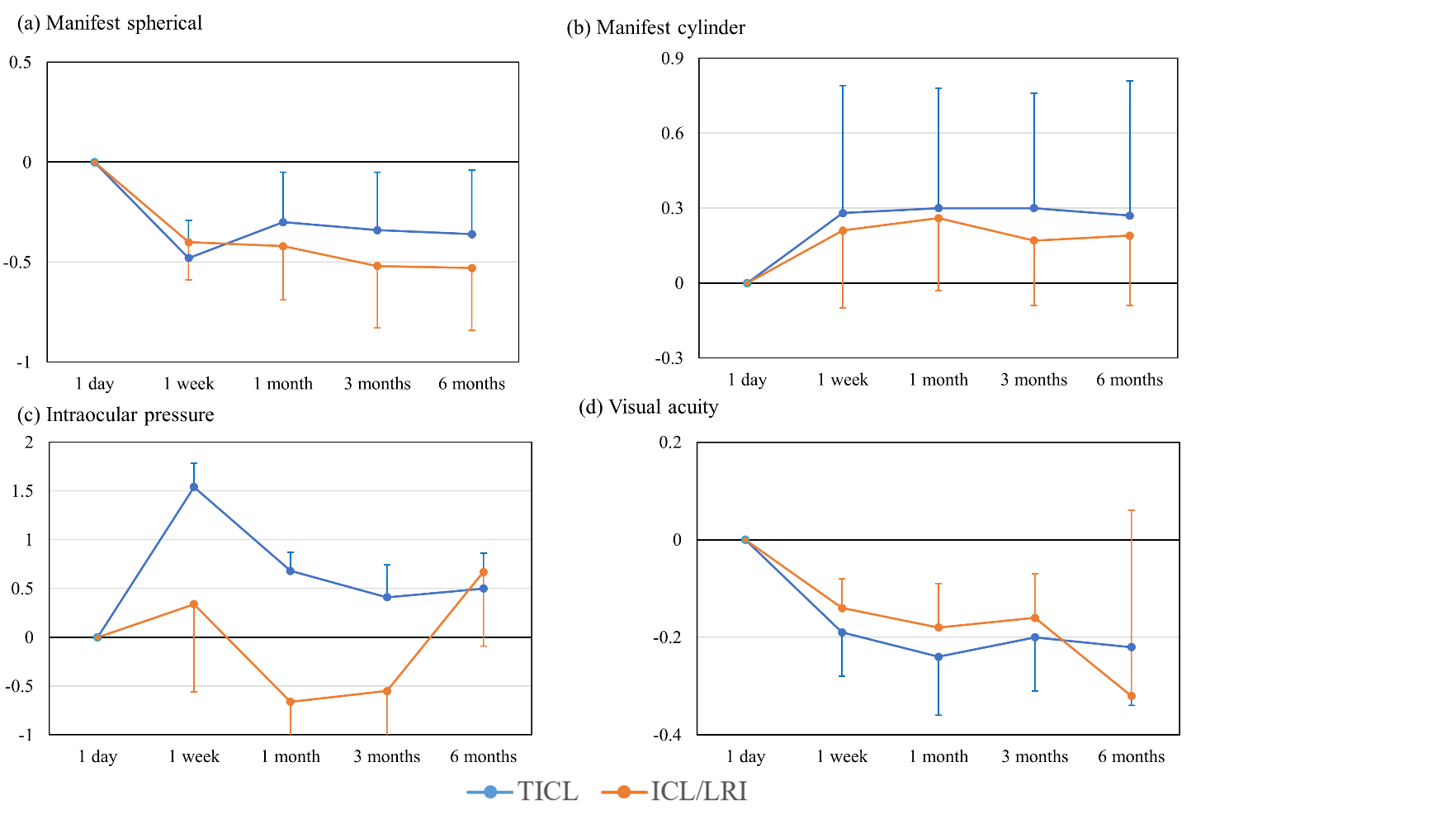


**Supplement Table contents**

Supplemental Table S1. Comparison of postoperative outcomes between ICL/LRI and TICL groups

| Variable | Total | Surgery | | |
| --- | --- | --- | --- | --- |
|  |  | ICL/LRI  (n=40) | TICL  (n=40) | *p*-value |
| Manifest spherical |  |  |  |  |
| 1 day | 0.75 (0.50, 1.25) | 0.75 (0.25, 1.00) | 1.00 (0.50, 1.25) | 0.247 |
| 1 week | 0.25 (0.00, 0.75) | 0.25 (-0.50, 0.50) | 0.50 (0.25, 0.75) | **0.006** |
| 1 month | 0.50 (0.25, 0.75) | 0.25 (0.00, 0.50) | 0.50 (0.25, 0.75) | **0.039** |
| 3 months | 0.25 (0.00, 0.50) | 0.25 (0.00, 0.50) | 0.50 (0.13, 0.50) | 0.096 |
| 6 months | 0.25 (0.00, 0.50) | 0.25 (0.00, 0.50) | 0.50 (0.25, 0.50) | 0.101 |
| Manifest cylinder |  |  |  |  |
| 1 day | -0.75 (-1.00, -0.50) | -0.75 (-1.00, -0.38) | -0.63 (-1.00, -0.50) | 0.949 |
| 1 week | -0.50 (-0.75, -0.25) | -0.50 (-0.50, -0.50) | -0.50 (-0.75, -0.25) | 1.000 |
| 1 month | -0.50 (-0.75, -0.25) | -0.50 (-0.75, -0.50) | -0.50 (-0.50, -0.25) | 0.143 |
| 3 months | -0.50 (-0.75, -0.25) | -0.50 (-0.75, -0.50) | -0.50 (-0.63, -0.25) | 0.558 |
| 6 months | -0.50 (-0.75, -0.50) | -0.50 (-0.75, -0.50) | -0.50 (-0.63, -0.50) | 0.380 |
| Intraocular pressure |  |  |  |  |
| 1 day | 15.00 (14.00, 17.00) | 15.00 (14.00, 17.00) | 15.00 (14.00, 16.50) | 0.623 |
| 1 week | 16.00 (15.00, 18.00) | 16.50 (14.00, 19.00) | 16.00 (15.50, 18.00) | 0.800 |
| 1 month | 15.00 (14.00, 17.00) | 16.00 (14.00, 18.00) | 15.00 (13.50, 16.50) | 0.056 |
| 3 months | 16.00 (14.00, 17.00) | 16.00 (14.00, 18.00) | 15.50 (15.00, 18.00) | 0.510 |
| 6 months | 16.00 (14.00, 18.00) | 15.00 (14.00, 17.00) | 16.00 (14.00, 17.00) | 0.375 |
| Visual acuity |  |  |  |  |
| 1 day | 0.00 (0.00, 0.22) | 0.00 (0.00, 0.22) | 0.00 (0.00, 0.11) | 0.654 |
| 1 week | -0.18 (-0.18, 0.00) | -0.18 (-0.18, 0.00) | -0.18 (-0.18, 0.00) | 0.718 |
| 1 month | -0.18 (-0.18, -0.18) | -0.18 (-0.18, 0.00) | -0.18 (-0.18, 0.00) | 0.961 |
| 3 months | -0.18 (-0.18, 0.00) | -0.18 (-0.18, 0.00) | -0.18 (-0.18, 0.00) | 0.864 |
| 6 months | -0.18 (-0.18, 0.00) | -0.18 (-0.18, 0.00) | -0.18 (-0.18, 0.00) | 0.545 |

Presented as median (IQR) and performed by Wilcoxon rank sum test. Significant values are in bold.

Supplemental Table S2. Comparison of postoperative outcomes between ICL/LRI and TICL groups in left eyes

| Variable | Total | Surgery | | |
| --- | --- | --- | --- | --- |
|  |  | ICL/LRI  (n=18) | TICL  (n=22) | *p*-value |
| Manifest spherical |  |  |  |  |
| 1 day | 1.00 (0.50, 1.00) | 1.00 (0.50, 1.00) | 1.00 (0.50, 1.00) | 1.000 |
| 1 week | 0.25 (0.00, 0.75) | 0.13 (-0.50, 0.63) | 0.50 (0.25, 0.75) | 0.120 |
| 1 month | 0.50 (0.25, 0.75) | 0.25 (0.00, 0.75) | 0.50 (0.25, 0.75) | 0.109 |
| 3 months | 0.50 (0.00, 0.50) | 0.25 (0.00, 0.50) | 0.50 (0.00, 0.50) | 0.191 |
| 6 months | 0.25 (0.00, 0.50) | 0.25 (0.00, 0.50) | 0.50 (0.00, 0.50) | 0.319 |
| Manifest cylinder |  |  |  |  |
| 1 day | -0.75 (-1.00, -0.50) | -0.63 (-1.00, -0.50) | -0.75 (-1.00, -0.50) | 0.781 |
| 1 week | -0.50 (-0.75, -0.25) | -0.50 (-0.50, -0.25) | -0.50 (-0.75, -0.25) | 0.397 |
| 1 month | -0.50 (-0.75, -0.50) | -0.50 (-0.75, -0.50) | -0.50 (-0.75, -0.25) | 0.647 |
| 3 months | -0.50 (-0.63, -0.25) | -0.50 (-0.75, -0.50) | -0.50 (-0.50, -0.25) | 0.663 |
| 6 months | -0.50 (-0.75, -0.50) | -0.50 (-0.75, -0.50) | -0.50 (-0.75, -0.50) | 0.784 |
| Intraocular pressure |  |  |  |  |
| 1 day | 15.00 (14.00, 16.00) | 15.00 (14.00, 17.00) | 15.00 (14.00, 16.00) | 0.640 |
| 1 week | 16.00 (15.00, 18.00) | 16.00 (14.00, 19.00) | 16.00 (16.00, 18.00) | 0.376 |
| 1 month | 15.50 (13.50, 17.00) | 16.00 (15.00, 18.00) | 15.00 (13.00, 17.00) | 0.221 |
| 3 months | 16.00 (15.00, 17.50) | 15.50 (14.00, 18.00) | 16.00 (15.00, 16.00) | 0.923 |
| 6 months | 15.50 (14.50, 17.50) | 16.50 (15.00, 18.00) | 15.00 (14.00, 16.00) | 0.071 |
| Visual acuity |  |  |  |  |
| 1 day | 0.00 (0.00, 0.22) | 0.00 (0.00, 0.22) | 0.00 (0.00, 0.22) | 0.951 |
| 1 week | -0.18 (-0.18, 0.00) | -0.18 (-0.18, 0.00) | -0.18 (-0.18, 0.00) | 0.384 |
| 1 month | -0.18 (-0.18, 0.00) | -0.18 (-0.18, 0.00) | -0.18 (-0.18, 0.00) | 0.478 |
| 3 months | -0.18 (-0.18, 0.00) | -0.18 (-0.18, 0.00) | -0.18 (-0.18, 0.00) | 0.829 |
| 6 months | -0.18 (-0.18, 0.00) | -0.18 (-0.18, 0.00) | -0.18 (-0.18, 0.00) | 0.608 |

Presented as median (IQR) and performed by Wilcoxon rank sum test. Significant values are in bold.

Supplemental Table S3. Comparison of postoperative outcomes between ICL/LRI and TICL groups in right eyes

| Variable | Total | Surgery | | |
| --- | --- | --- | --- | --- |
|  |  | ICL/LRI  (n=22) | TICL  (n=18) | *p*-value |
| Manifest spherical |  |  |  |  |
| 1 day | 0.75 (0.25, 1.25) | 0.50 (0.25, 1.00) | 0.75 (0.25, 1.50) | 0.177 |
| 1 week | 0.25 (0.00, 0.63) | -0.25 (0.25, 0.25) | 0.50 (0.25, 0.75) | **0.019** |
| 1 month | 0.50 (0.25, 0.50) | 0.50 (0.00, 0.50) | 0.25 (0.50, 0.75) | 0.250 |
| 3 months | 0.25 (0.00, 0.50) | 0.25 (0.00, 0.50) | 0.38 (0.25, 0.50) | 0.325 |
| 6 months | 0.25 (0.00, 0.50) | 0.25 (0.00, 0.50) | 0.50 (0.25, 0.50) | 0.204 |
| Manifest cylinder |  |  |  |  |
| 1 day | -0.50 (-1.00, -0.38) | -0.75 (-1.00, -0.25) | -0.50 (-0.75, -0.50) | 0.658 |
| 1 week | -0.50 (-0.50, -0.50) | -0.50 (-0.75, -0.50) | -0.50 (-0.50, -0.25) | 0.328 |
| 1 month | -0.50 (-0.50, -0.25) | -0.50 (-0.75, -0.50) | -0.50 (-0.50, -0.25) | 0.075 |
| 3 months | -0.50 (-0.75, -0.25) | -0.50 (-0.75, -0.50) | -0.50 (-0.75, -0.25) | 0.700 |
| 6 months | -0.50 (-0.75, -0.50) | -0.50 (-0.75, -0.50) | -0.50 (-0.50, -0.25) | 0.238 |
| Intraocular pressure |  |  |  |  |
| 1 day | 15.00 (14.00, 17.00) | 15.00 (14.00, 17.00) | 15.00 (14.00, 17.00) | 0.901 |
| 1 week | 16.00 (15.00, 18.50) | 17.00 (15.00, 19.00) | 16.00 (15.00, 17.00) | 0.225 |
| 1 month | 15.00 (14.00, 17.00) | 17.00 (14.00, 18.00) | 15.00 (14.00, 16.00) | 0.178 |
| 3 months | 15.00 (14.00, 17.00) | 16.00 (14.00, 18.00) | 15.00 (14.00, 16.00) | 0.370 |
| 6 months | 14.00 (16.00, 18.00) | 15.00 (14.00, 17.00) | 16.00 (14.00, 18.00) | 0.509 |
| Visual acuity |  |  |  |  |
| 1 day | 0.00 (0.00, 0.11) | 0.00 (0.00, 0.22) | 0.00 (0.00, 0.00) | 0.545 |
| 1 week | -0.18 (-0.18, 0.00) | -0.18(-0.18, 0.00) | -0.09 (-0.18, 0.00) | 0.667 |
| 1 month | -0.18 (-0.18, 0.00) | -0.18 (-0.18, -0.18) | -0.18 (-0.18, 0.00) | 0.529 |
| 3 months | -0.18 (-0.18, 0.00) | -0.18 (-0.18, 0.00) | -0.18 (-0.18, 0.00) | 0.925 |
| 6 months | -0.18 (-0.18, 0.00) | -0.18 (-0.18, 0.00) | -0.18 (-0.18, 0.00) | 0.606 |

Presented as median (IQR) and performed by Wilcoxon rank sum test. Significant values are in bold.

Supplement Table S4. The association between the surgery and the postoperative performance of manifest spherical, manifest cylinder, intraocular pressure, and visual acuity in left eyes.

| Variable | Manifest sphere | |  | Manifest cylinder | |  | Intraocular pressure | |  | Visual acuity | |
| --- | --- | --- | --- | --- | --- | --- | --- | --- | --- | --- | --- |
|  | β (95% CI) | *p*-value |  | β (95% CI) | *p*-value |  | β (95% CI) | *p*-value |  | β (95% CI) | *p*-value |
| Age | -0.14 (-0.42, 0.15) | 0.352 |  | -0.01 (-0.01, 0.00) | 0.090 |  | 0.25 (-0.03, 0.54) | 0.083 |  | 0.01 (0.00, 0.01) | 0.089 |
| Male (vs. female) | 1.75 (-1.12, 4.61) | 0.232 |  | -0.11 (-0.24, 0.01) | 0.069 |  | -1.30 (-4.33, 1.72) | 0.400 |  | 0.03 (-0.10, 0.16) | 0.652 |
| Surgery (vs. TICL) |  |  |  |  |  |  |  |  |  |  |  |
| ICL/LRI | 0.07 (-0.87, 1.00) | 0.889 |  | -0.03 (-0.24, 0.19) | 0.798 |  | -1.43 (-3.72, 0.86) | 0.22 |  | -0.01 (-0.13, 0.11) | 0.906 |
| Time (vs. 1 day) |  |  |  |  |  |  |  |  |  |  |  |
| 1 week | -0.81 (-1.17, -0.44) | **<0.001** |  | **0.29 (0.06, 0.52)** | **0.012** |  | 0.13 (-0.74, 1.00) | 0.768 |  | -0.17 (-0.24, -0.09) | **<0.001** |
| 1 month | -0.53 (-0.75, -0.30) | **<0.001** |  | 0.19 (-0.06, 0.44) | 0.142 |  | 0.48 (-0.22, 1.19) | 0.180 |  | -0.21 (-0.31, -0.12) | **<0.001** |
| 3 months | -0.56 (-0.80, -0.32) | **<0.001** |  | 0.24 (-0.03, 0.50) | 0.079 |  | 0.52 (-0.39, 1.43) | 0.260 |  | -0.23 (-0.32, -0.14) | **<0.001** |
| 6 months | -0.58 (-0.83, -0.33) | **<0.001** |  | 0.17 (-0.10, 0.44) | 0.214 |  | **1.17 (0.11, 2.23)** | **0.031** |  | -0.20 (-0.32, -0.09) | **<0.001** |
| Time x Surgery (vs. 1 day, TICL) |  |  |  |  |  |  |  |  |  |  |  |
| 1 week | **0.42 (0.03, 0.81)** | **0.037** |  | -0.07 (-0.28, 0.14) | 0.533 |  | **1.66 (0.27, 3.04)** | **0.019** |  | 0.00 (-0.12, 0.11) | 0.962 |
| 1 month | **0.32 (0.03, 0.61)** | **0.031** |  | 0.06 (-0.17, 0.30) | 0.600 |  | -0.50 (-2.07, 1.07) | 0.534 |  | 0.01 (-0.11, 0.13) | 0.926 |
| 3 months | 0.10 (-0.19, 0.40) | 0.490 |  | 0.03 (-0.23, 0.30) | 0.796 |  | 0.36 (-1.13, 1.86) | 0.634 |  | 0.03 (-0.09, 0.15) | 0.595 |
| 6 months | 0.08 (-0.22, 0.37) | 0.606 |  | 0.04 (-0.25, 0.33) | 0.77 |  | -1.08 (-2.67, 0.51) | 0.185 |  | 0.01 (-0.12, 0.15) | 0.860 |

Presented as β (95%CI) and performed by generalized estimating equations (GEE) with unstructured matrix. Significant values are in bold.

Supplement Table S5. The association between the surgery and the postoperative performance of manifest spherical, manifest cylinder, intraocular pressure, and visual acuity in right eyes.

| Variable | Manifest spherical | |  | Manifest cylinder | |  | Intraocular pressure | | |  | | Visual acuity | |
| --- | --- | --- | --- | --- | --- | --- | --- | --- | --- | --- | --- | --- | --- |
|  | β (95% CI) | *p*-value |  | β (95% CI) | *p*-value |  | β (95% CI) | *p*-value |  | | β (95% CI) | | *p*-value |
| Age | 0.00 (-0.05, 0.06) | 0.912 |  | -0.03 (-0.05, 0.00) | 0.071 |  | -0.21 (-0.44, 0.02) | 0.075 |  | | 0.03 (-0.01, 0.07) | | 0.198 |
| Male (vs. female) | 0.15 (-0.69, 0.99) | 0.728 |  | **0.61 (0.01, 1.21)** | **0.047** |  | 1.09 (-0.16, 2.34) | 0.088 |  | | 0.07 (-0.21, 0.35) | | 0.609 |
| Surgery (vs. TICL) |  |  |  |  |  |  |  |  |  | |  | |  |
| ICL/LRI | 0.19 (-0.18, 0.57) | 0.313 |  | 0.13 (-0.23, 0.49) | 0.476 |  | 0.08 (-1.65, 1.81) | 0.931 |  | | 0.00 (-0.14, 0.14) | | 0.991 |
| Time (vs. 1 day) |  |  |  |  |  |  |  |  |  | |  | |  |
| 1 week | **-0.58 (-0.84, -0.32)** | **<0.001** |  | 0.32 (-0.06, 0.71) | 0.097 |  | **1.67 (0.59, 2.75)** | **0.003** |  | | -0.17 (-0.23, -0.11) | | **<0.001** |
| 1 month | **-0.29 (-0.53, -0.05)** | **0.019** |  | 0.26 (-0.21, 0.74) | 0.275 |  | 0.82 (-0.19, 1.82) | 0.114 |  | | -0.22 (-0.30, -0.14) | | **<0.001** |
| 3 months | **-0.33 (-0.55, -0.10)** | **0.005** |  | **0.41 (0.02, 0.81)** | **0.040** |  | 0.47 (-0.71, 1.66) | 0.435 |  | | -0.20 (-0.29, -0.11) | | **<0.001** |
| 6 months | **-0.34 (-0.57, -0.11)** | **0.004** |  | **0.39 (0.02, 0.75)** | **0.040** |  | 0.56 (-0.56, 1.68) | 0.328 |  | | -0.22 (-0.32, -0.12) | | **<0.001** |
| Time x Surgery (vs. 1 day, TICL) |  |  |  |  |  |  |  |  |  | |  | |  |
| 1 week | 0.20 (-0.16, 0.56) | 0.270 |  | -0.13 (-0.52, 0.27) | 0.531 |  | -1.08 (-2.50, 0.34) | 0.135 |  | | 0.02 (-0.09, 0.13) | | 0.767 |
| 1 month | -0.08 (-0.39, 0.24) | 0.633 |  | -0.01 (-0.52, 0.50) | 0.963 |  | -1.22 (-2.51, 0.07) | 0.064 |  | | 0.01 (-0.11, 0.14) | | 0.853 |
| 3 months | -0.14 (-0.47, 0.19) | 0.404 |  | -0.27 (-0.65, 0.10) | 0.147 |  | -0.88 (-2.66, 0.90) | 0.333 |  | | 0.00 (-0.13, 0.12) | | 0.961 |
| 6 months | -0.13 (-0.49, 0.23) | 0.472 |  | -0.20 (-0.53, 0.14) | 0.245 |  | 0.24 (-1.59, 2.07) | 0.795 |  | | -0.05 (-0.22, 0.12) | | 0.565 |

Presented as β (95%CI) and performed by generalized estimating equations (GEE) with unstructured matrix. Significant values are in bold.
